# Changes in the lifestyle behavior and anthropometrics of university students after the first year: a one-year prospective observational study

**DOI:** 10.3389/fspor.2025.1499828

**Published:** 2025-05-20

**Authors:** Héctor Gutiérrez-Espinoza, María Cassola-Cajiao, Emilia Garzón-Ulloa, Daniela Celi-Lalama, Felipe Araya-Quintanilla, Juan Valenzuela-Fuenzalida, José Francisco López-Gil

**Affiliations:** ^1^ Faculty of Education, Universidad Autónoma de Chile, Santiago, Chile; ^2^ Escuela de Fisioterapia, Universidad de las Americas, Quito, Ecuador; ^3^ School of Physiotherapy, Faculty of Medical, Health and Life Sciences, International University of Ecuador, UIDE, Quito, Ecuador; ^4^ Escuela de Kinesiología, Facultad de Odontología y Ciencias de la Rehabilitación, Universidad San Sebastián, Santiago, Chile; ^5^ Departamento de Morfología, Facultad de Medicina, Universidad Andrés Bello, Santiago, Chile; ^6^ School of Medicine, Universidad Espíritu Santo, Samborondón, Ecuador; ^7^ Vicerrectoría de Investigación y Postgrado, Universidad de Los Lagos, Osorno, Chile

**Keywords:** lifestyle behavior, anthropometry, sleep quality, 24-hour movement guidelines, university students, observational study

## Abstract

**Background:**

The first year of university has been identified as a period of adoption of unhealthy lifestyle behaviors. However, only a few studies have addressed the extent of this phenomenon in Latin American universities. The aim of this study was to examine changes in physiotherapy students' lifestyle behaviors after one year at university.

**Methods:**

A total of 100 students of *Universidad de Las Americas* in Quito, Ecuador, were prospectively recruited. In all patients' sociodemographic variables, anthropometric indices, adherence to 24-hour movement guidelines (i.e., physical activity, sedentary behavior, and sleep), diet, and alcohol consumption were assessed. Measurements were performed at the start of the first academic semester and after one year at university.

**Results:**

The total of sample, 60 students were female (60%), 40 were male (40%), and the mean age was 21.2 years old. At one-year follow-up, the mean difference (MD) for weight was +5.8 kg [95% confidence interval (CI): 2.9 to 8.5; *p* < 0.001] and for body mass index was +3.3 kg/m^2^ (95% CI: 1.1 to 5.2; *p* = 0.001). Additionally, number of students with high levels of physical activity according to the Global Physical Activity Questionnaire (GPAQ) decreased from 42 to 28 (*p* < 0.001), the MD for total metabolic equivalents (METs) per week was −1.0 (95% CI −0.8 to −2.0; *p* = 0.001), and decreased from 10 to 6 students (*p* = 0.042) who met the overall 24-hour movement guidelines. Finally, there was an increase from 70 to 79 students (*p* = 0.041) who showed significant sleep disturbances, from 70 to 80 students (*p* = 0.035) who needed changes in diet quality, and from 20 to 30 students (*p* = 0.035) who showed harmful alcohol consumption.

**Conclusions:**

At the end of the first year of university, most students showed unhealthy lifestyle behaviors characterized by an increase in weight and body mass index, a decrease in the level of physical activity, low adherence to 24-hour movement guidelines, more sleep disturbance, poor diet quality, and harmful alcohol consumption. This indicates a need to address this problem during this critical period for developing interventions to improve lifestyle behaviors and prevent the occurrence of non-communicable chronic diseases.

## 1 Introduction

University presents a time of transition for young adults that involves behavior adaptation in a new environment, with new relationships and changing support systems (1). University students are in a period of transition to adulthood marked by important lifestyle changes (2, 3). Indeed, when analyzing patterns of lifestyle change over the lifespan, a critical time for negative lifestyle behaviors appears to be in later adolescence and early adulthood (4–6). In this sense, the first year of university is a critical period because it is associated with the adoption of unhealthy lifestyle behaviors, where students experience changes in eating behaviors, physical activity, sleep patterns, and alcohol consumption (7–9).

Consequently, several meta-analyses have reported that the first year of university is accompanied by important changes in lifestyle behavior characterized by an increase in weight and percentage of body fat, changes in diet quality or eating behaviors, and decreased levels of physical activity (2, 8, 10–13). The main behaviors associated with weight gain were the consumption of sugary drinks, alcohol consumption, low consumption of vegetables and fruits, frequent self-consumption, eating unhealthy and low levels of food regulation (13). Moreover, a global survey of 23 countries with different income levels showed that 21.9% to 80.6% of university students are physically inactive (14). On the other hand, the transition to university life often leads students to modify their dietary habits, sleep patterns, and alcohol consumption due to increased autonomy, academic pressures, and new social environments (2, 12, 15).

According to the World Health Organization (WHO), a healthy lifestyle involves regular physical exercise, abstention from smoking, avoiding alcohol consumption, and eating healthy foods to prevent excess body weight gain (16). Currently, evidence supports that aspects of movement behavior such as levels of physical activity, sedentary time, and duration of sleep are interconnected as they occur throughout the 24-hour day, and thus, they should be assessed collectively (17, 18). In line with this, in a previous cross-sectional study, we reported that upon admission to university, overall adherence to the 24-hour movement guidelines is low, and a high percentage of physiotherapy students reported unhealthy lifestyle behaviors in Ecuador (6).

Several studies have analyzed changes in lifestyle behaviors at one or more years of follow-up in university students (5, 7, 19–22). However, only one published study has been performed in Latin American university students (9). Given the scarcity of published data about the behavior of these variables in this population. Thus, the aim of this study was to examine changes in lifestyle behavior, such as physical activity, sleep quality and duration, adherence to 24-hour movement guidelines, diet, and alcohol consumption in physiotherapy students after one year in the *Universidad de Las Americas* in Quito, Ecuador.

## 2 Materials and methods

### 2.1 Design/ethics

This prospective observational study was conducted following the Strengthening the Re­porting of Observational studies in Epidemiology (STROBE) guidelines (23). The study was approved by the Ethics Committee for Research on Human Beings of *Universidad de Las Americas* (Ecuador) (ID: 2022-OBS-008) and received funding from Universidad de Las Americas with sponsor code FIS.HGE.23.01. In the second semester of 2022 and the first semester of 2023, all students entering the physiotherapy school were prospectively recruited (116 students). All participants signed an informed consent form approved by the ethics committee.

### 2.2 Participants

We included all students from 18 years old who entered physiotherapy school at the *Universidad de Las Americas* in the city of Quito, Ecuador, during two academic semesters and who were current students of the program at one-year follow-up. Conversely, students were excluded if they had the following conditions: (i) any uncontrolled comorbidities, such as hypertension, diabetes mellitus, or hypercholesterolemia; or (ii) physical impairments associated with neurological diseases or mental illness.

### 2.3 Outcomes measures

Two evaluators from the scientific staff assessed the variables at the start of the first academic semester and after one year at university. Before the study began, these evaluators with postgraduate qualifications in Kinanthropometry received two weeks of training to standardize the measurements. Both assessed the same proportion of participants.

#### 2.3.1 Sociodemographic and comorbidity variables

Age, sex, socioeconomic status (high, middle, or low), and work occupation parallel to study; middle-time (10 to 22 hours per week), part-time (less than 10 hours per week), or not working were assessed for each participant. Comorbidity data, including hypertension (defined as a systolic blood pressure ≥140 mmHg, diastolic blood pressure 90 mmHg, or receiving treatment with an antihypertensive agent), diabetes mellitus (defined as a fasting plasma glucose ≥ 126 mg/dl, Glycated Hemoglobin A1c > 6.5%, or treatment with a hypoglycemic agent or insulin) and hypercholesterolemia (defined as levels of low-density lipoprotein cholesterol >130 mg/dl), were also assessed.

#### 2.3.2 Anthropometric variables

Participants had their waist circumference measured in a bipedal position using a non-elastic tape placed midway between the costal margins and the iliac crest in the horizontal plane. Height was measured barefoot using a Seca 213 stadiometer, ensuring the sagittal midline touched the backboard. Body weight, measured with a Tanita HD-351, was taken with participants barefoot and in light clothing. These measurement procedures align with the recommendations of the International Society for the Advancement of Kinanthropometry. Finally, body mass index (BMI) was computed as the weight in kilograms divided by the square of height in meters, and the BMI values were used to determine nutritional status following the WHO standards (24).

#### 2.3.3 Physical activity

The Global Physical Activity Questionnaire (GPAQ) was used to assess the level of physical activity (20). The GPAQ is a validated 16-item scale that measures moderate- and vigorous-intensity physical activity in three separate domains such as work, recreation, and active transportation domains during a typical week (25). The GPAQ has been established as a valid and reliable instrument to measure physical activity among university students (26).

#### 2.3.4 Sleep quality and duration

The assessment of sleep quality and duration utilized the Pittsburgh Sleep Quality Index (PSQI) questionnaire (27). This self-rated questionnaire consists of 19 items and evaluates sleep quality over a one-month period. The total score, ranging from 0 to 21, indicates sleep quality, with higher scores suggesting poorer sleep quality. A total score exceeding five points indicates significant sleep disturbance (27). The Spanish version of the PSQI is recognized as a valid and reliable instrument for measuring sleep quality (28).

#### 2.3.5 Adherence to 24-hour movement guidelines

To assess adherence to the Canadian 24-hour movement guidelines for adults, participants must meet the following conditions: (a) engage in at least 150 min per week of moderate-to-vigorous physical activity, incorporating muscle-strengthening activities at least twice a week; (b) limit sedentary time to no more than eight hours daily and recreational screen time to no more than three hours daily; and (c) maintain a nightly sleep duration of seven to nine hours (17). Based on data obtained from personal assessments and two self-report questionnaires (25, 27), participants meeting all three criteria were categorized as compliant with the guidelines (17).

#### 2.3.6 Diet

Diet quality was assessed with the Spanish version of the Healthy Eating Index (HEI) (29). Participants' diets were classified according to the total score and divided into three categories: >80 points, “*healthy*”; 50–80 points, “*needs changes*”; and <50 points, “*unhealthy*”. This is a valid and reliable instrument for measuring dietary quality (30).

#### 2.3.7 Alcohol consumption

Alcohol consumption was assessed with the Alcohol Use Disorders Identification Test (AUDIT) (31). This questionnaire is specifically designed to assess hazardous alcohol consumption. Scores on the AUDIT range from 0 to 40, with higher scores indicative of an elevated risk of problematic alcohol consumption (31). A total score exceeding eight points is considered a cutoff point indicating harmful alcohol consumption (32). The Ecuadorian adaptation of the Spanish version of the AUDIT has demonstrated good internal consistency and reliability (33).

### 2.4 Statistical analysis

Descriptive statistics were used to characterize the sociodemographic, anthropometric, and lifestyle variables of the participants. Continuous variables are presented as the mean and standard deviation (SD), and categorical variables are presented as the number and percentage. To determine the statistical tests for use in data analysis, we used Shapiro–Wilk statistical test. For continuous variables, a paired *t*-test was used to determine the differences between the data at baseline and one-year follow-up, and the McNemar test was used for categorical variables. Statistical significance was established at *p* < 0.05. Statistical analysis was performed using Stata 16.0 (Stata, College Station, TX, USA).

## 3 Results

**Figure 1:**
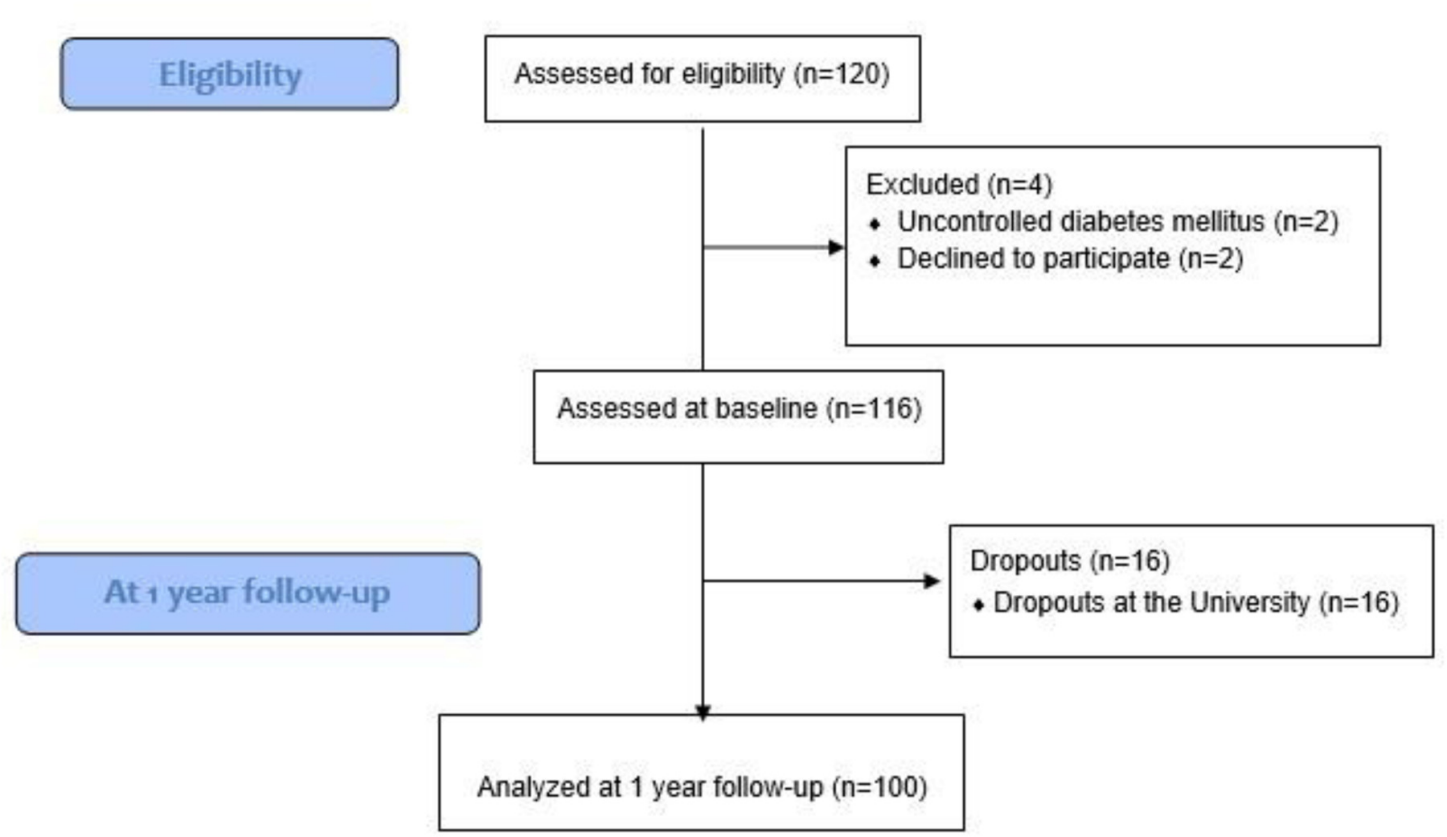
Flow chart diagram of participants in the study.

Of the 120 students considered for eligibility, 2 students were excluded due to uncontrolled diabetes mellitus, and 2 declined participation. Finally, 116 students were initially recruited; however, 16 dropped out during their first year of University. Therefore, 100 students were included in this study (Figure 1). Characteristics, sociodemographics, and anthropometrics of the students included are shown in Table 1. Of these 100 university students included; 60 were female (60%), 40 were male (40), and the mean age was 21.2 years old (SD = 1). A total of 70 students (70%) reported middle socioeconomic status, and 80 (80%) were not working. Additionally, 80 students (80%) did not present any diagnosed comorbidity (hypertension, diabetes mellitus or hypercholesterolemia). According to stratified BMI, 40 students (40%) had normal weight, 40 (40%) were overweight, and 19 (19%) had obesity.

**Table 1:** Sociodemographic data, medical conditions, and anthropometric of the study sample.

Variables	Total (*N* = 100)	Males (*n* = 40)	Females (*n* = 60)
Sociodemographic information			
Age (y), mean (SD)	21.2 (1.0)	23.2 (0.8)	19.2 (1.2)
Socioeconomic status, number (%)			
– High	30.0 (30.0)	12.0 (12.0)	18.0 (18.0)
– Middle	70.0 (70.0)	28.0 (28.0)	42.0 (42.0)
– Low	–	–	–
Work occupation, number (%)			
– Middle time	–	–	–
– Part time	20.0 (20.0)	12.0 (12.0)	8.0 (8.0)
– Not working	80.0 (80.0)	28.0 (28.0)	52.0 (52.0)
Comorbidities (hypertension, diabetes mellitus, hypercholesterolemia), number (%)			
– None	80.0 (80.0)	38.0 (38.0)	42.0 (42.0)
– Only one	19.0 (19.0)	1.0 (1.0)	18.0 (18.0)
– Two	1.0 (1.0)	1.0 (1.0)	–
– All three	–	–	–
Anthropometric information			
Weight (kg), mean (SD)	61.7 (10.9)	69.7 (11.3)	53.7 (10.5)
Height (cm), mean (SD)	163.0 (0.5)	167.0 (0.5)	159.0 (0.5)
Stratified BMI, number (%)			
– Underweight (<18.5)	1.0 (1.0)	–	1.0 (1.0)
– Normal (18.5–24.9)	40.0 (40.0)	13.0 (13.0)	27.0 (27.0)
– Overweight (25–29.9)	40.0 (40.0)	20.0 (20.0)	20.0 (20.0)
– Obesity (30–39.9)	19.0 (19.0)	7.0 (7.0)	12.0 (12.0)
– Extreme Obesity (>40)	–	–	–

BMI, body mass index; SD, standard deviation.

Additionally, changes in the continuous anthropometrics and lifestyle variables between the baseline and one-year follow-up are shown in Table 2. The mean difference (MD) for weight was +5.8 kg [95% confidence interval (CI): 2.9 to 8.5; *p* < 0.001] and for BMI was +3.3 kg/m^2^ (95% CI: 1.1 to 5.2; *p* = 0.001). The MDs for PSQI and AUDIT questionnaires increased by 1.0 point each (both *p*-values were less than 0.05).

**Table 2:** Changes in the continuous anthropometrics and lifestyle variables at 1-year follow-up.

Outcomes measures	Baseline	At 1-year follow-up	Mean difference (95% CI)	*p*-value* for normal distribution	*p*-value**
Weight (kg), mean (SD)	61.7 (10.9)	67.5 (8.1)	5.8 (2.9 to 8.5)	*p* = 0.73	***p*** **<** **0.001**
BMI (kg/m^2^), mean (SD)	23.9 (3.5)	27.2 (3.1)	3.3 (1.1 to 5.2)	*p* = 0.67	***p*** **=** **0.001**
Total METs (week), mean (SD)	4.2 (2.6)	3.2 (1.9)	−1.0 (−0.8 to −2)	*p* = 0.53	***p*** **=** **0.024**
Total PSQI (points), mean (SD)	7.2 (1.8)	8.2 (1.2)	1.0 (0.5 to 1.6)	*p* = 0.55	***p*** **=** **0.036**
Total AUDIT (points), mean (SD)	5.4 (3.2)	6.4 (2.4)	1.0 (0.6 to 1.7)	*p* = 0.35	***p*** **=** **0.029**

AUDIT, alcohol use disorders identification test; BMI, body mass index; CI, confidence interval; METs, metabolic equivalents; PSQI, pittsburgh sleep quality index; SD, standard deviation.

* *p*-value were obtained with Shapiro–Wilk test.

** Differences between baseline and 1-year follow-up were obtained with paired *t*-test. Bold indicates a *p*-value < 0.05.

**Figure 2:**
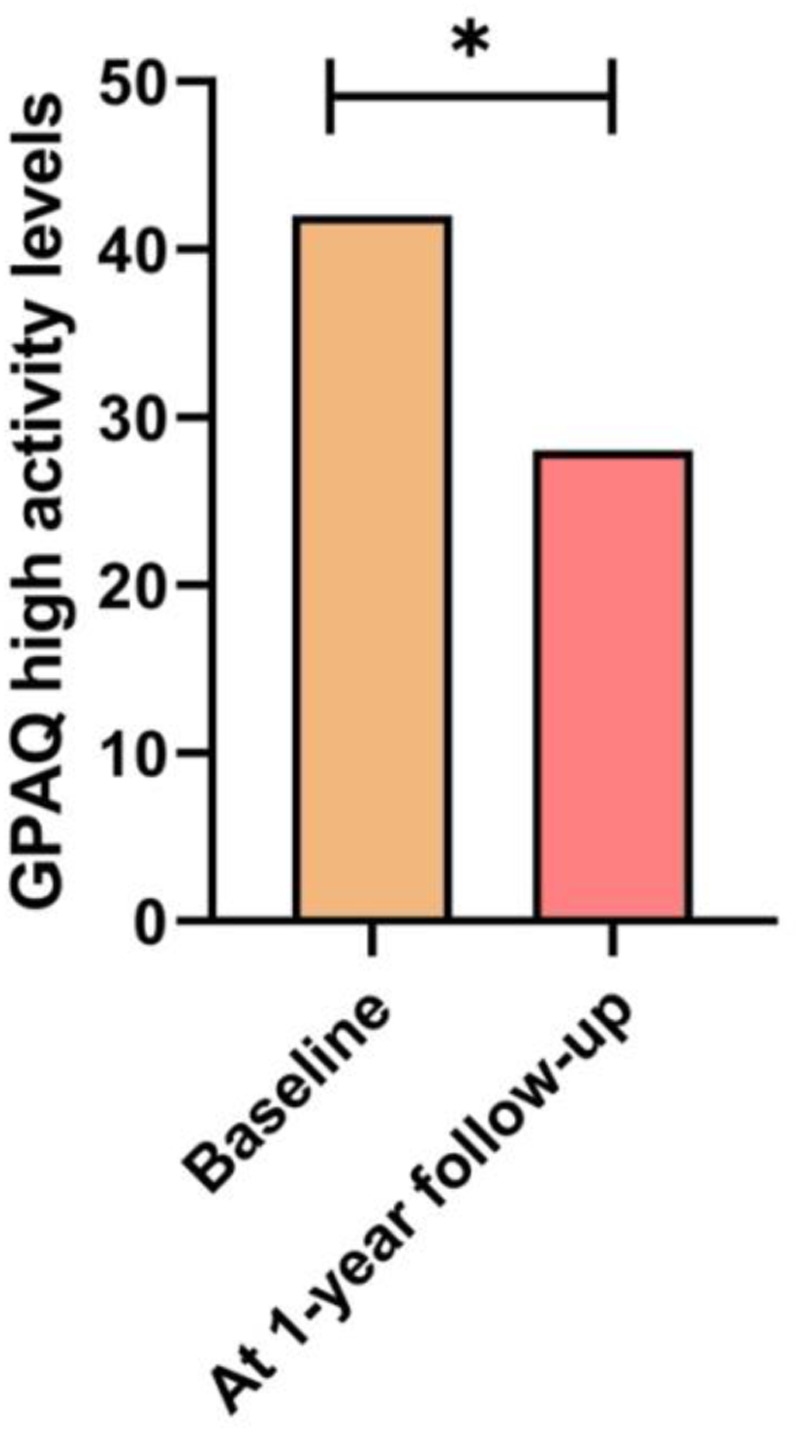
Changes in the number of students with high levels of physical activity according to GPAQ. *Statistically significant difference (*p* < 0.001).

**Figure 3:**
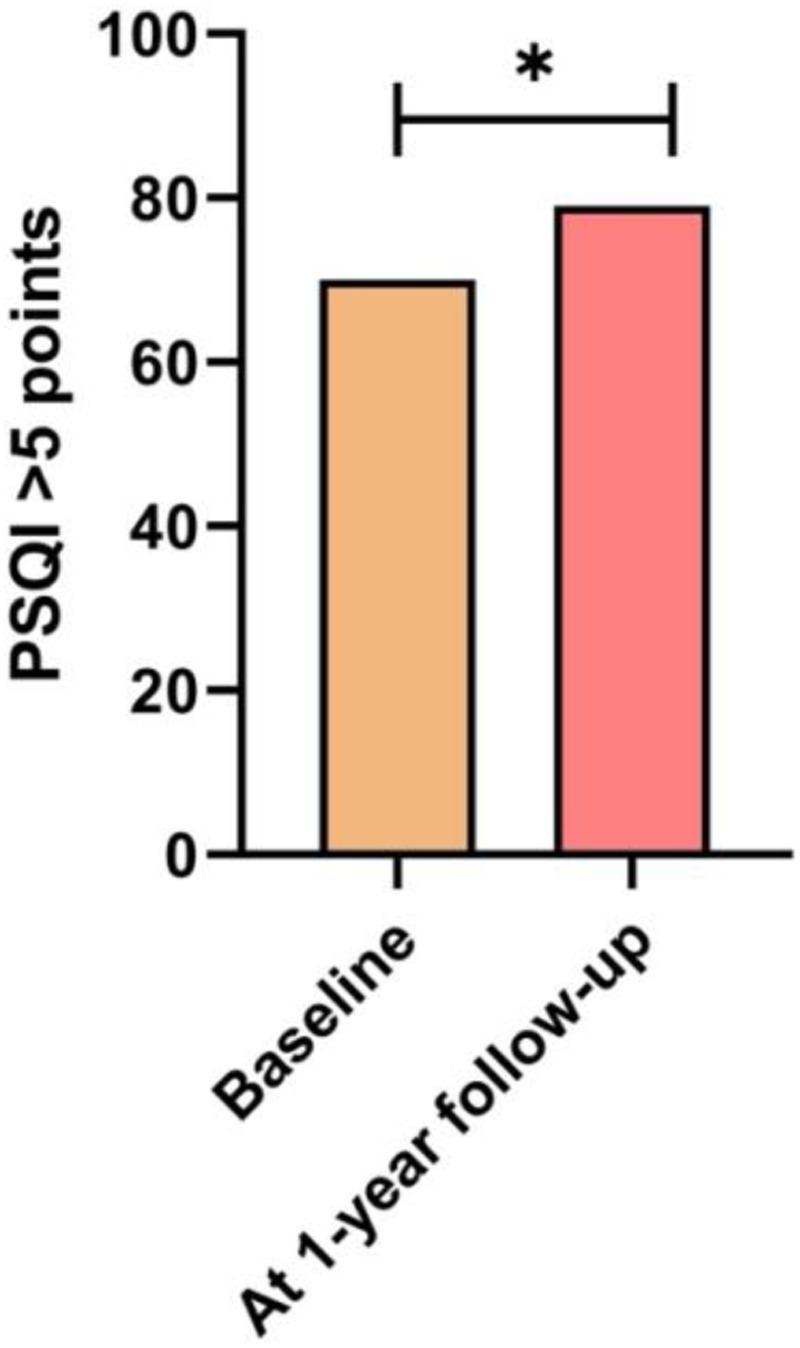
Changes in the number of students with significant sleep disturbances according to PSQI. *Statistically significant difference (*p* < 0.041).

**Figure 4:**
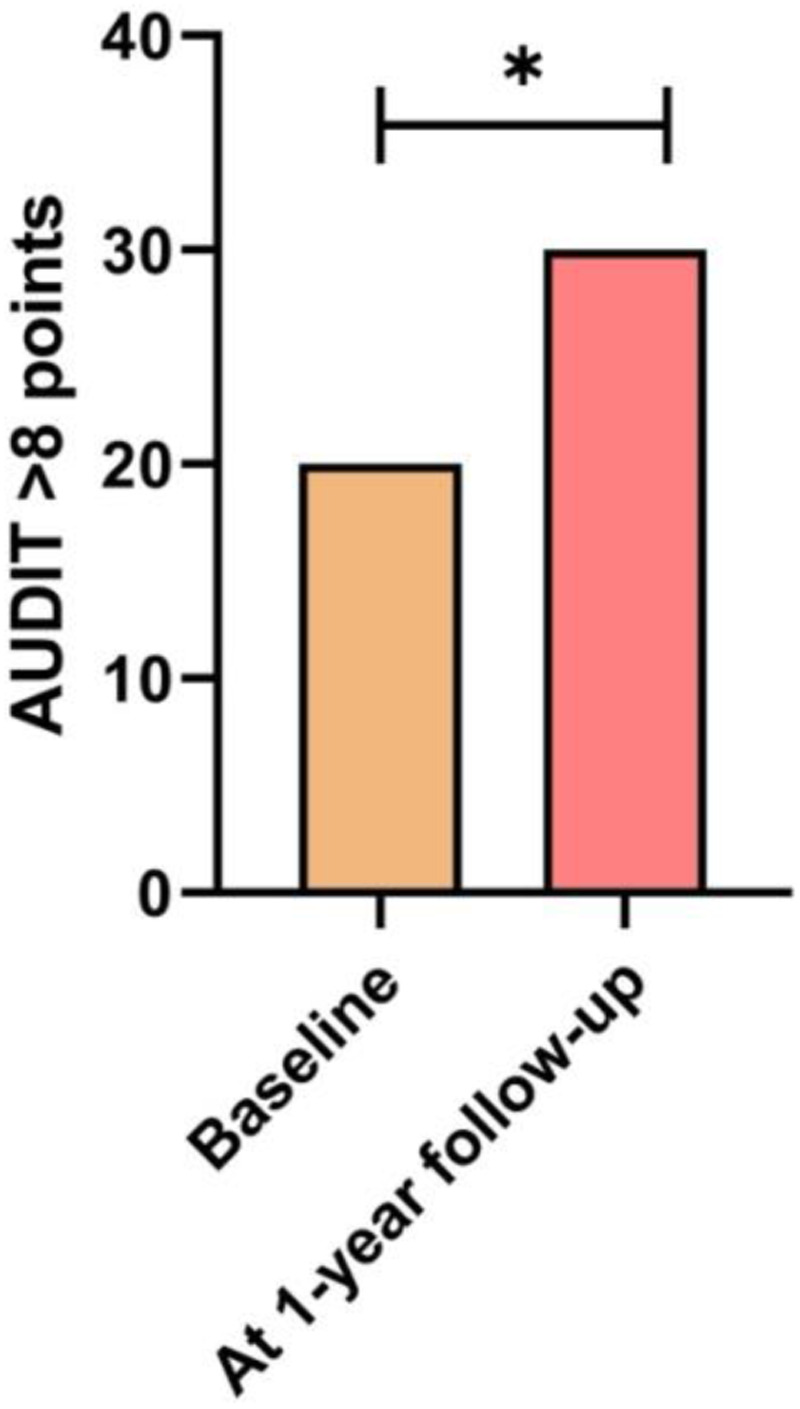
Changes in the number of students number of students with harmful alcohol use according to AUDIT. *Statistically significant difference (*p* < 0.035).

**Figure 5:**
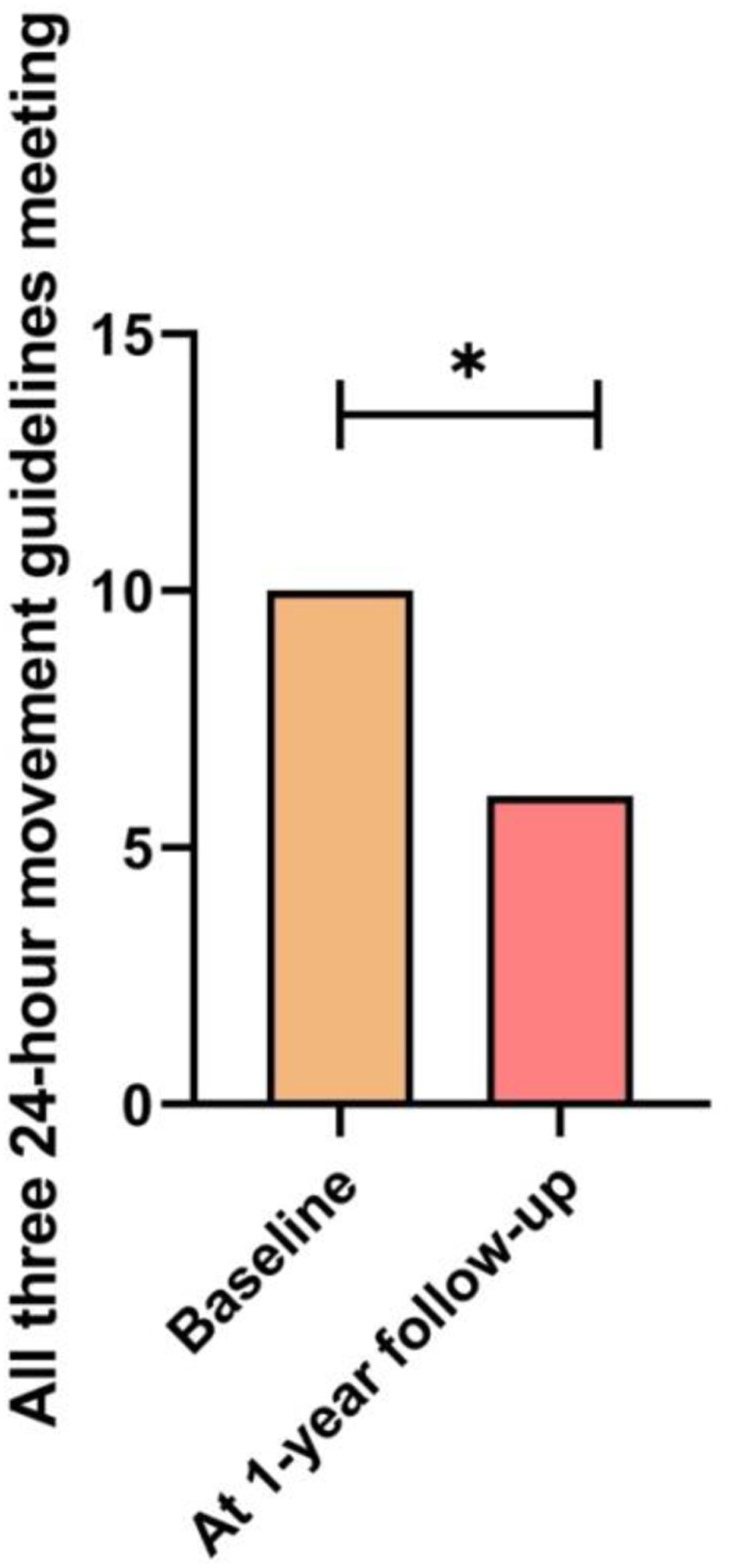
Changes in the number of students that meeting all recommendations of adherence to the overall 24-hour movement guidelines. *Statistically significant difference (*p* < 0.042).

Changes in categorical anthropometric and lifestyle variables between the baseline and one-year follow-up are shown in Table 3. Figure 2 shows the number of students with high levels of physical activity according to GPAQ, the Figure 3 shows the number of students experiencing significant sleep disturbances (PSQI > 5 points), the Figure 4 shows the number of students with harmful alcohol use (AUDIT > 8 points). Finally, the Figure 5 shows the number of students that meeting all recommendations of adherence to the overall 24-hour movement guidelines.

**Table 3:** Changes in the categorical anthropometrics and lifestyle variables at 1-year follow-up.

Outcomes measures	Baseline	At 1-year follow-up	*p*-value*
GPAQ activity levels, number (%)			
– High	42.0 (42.0)	28.0 (28.0)	***p*** **<** **0.001**
– Moderate	32.0 (32.0)	30.0 (30.0)	
– Low	26.0 (26.0)	42.0 (42.0)	
Students with PSQI > 5 points, number (%)	70.0 (70.0)	79.0 (79.0)	***p*** **=** **0.041**
PA recommendations meeting, number (%)	12.0 (12.0)	8.0 (8.0)	*p* = 0.067
Sedentary behavior meeting, number (%)	13.0 (13.0)	10.0 (10.0)	*p* = 0.072
Sleep duration meeting, number (%)	16.0 (16.0)	12.0 (12.0)	***p*** **=** **0.045**
All three 24-hour movement guidelines meeting, number (%)	10.0 (10.0)	6.0 (6.0)	***p*** **=** **0.042**
Categories according to HEI, number (%)			
– Healthy	20.0 (20.0)	6.0 (6.0)	***p*** **<** **0.001**
– Need change	70.0 (70.0)	80.0 (80.0)	
– Unhealthy	10.0 (10.0)	14.0 (14.0)	
Students with AUDIT > 8 points, number (%)	20.0 (20.0)	30.0 (30.0)	***p*** **=** **0.035**

AUDIT, alcohol use disorders identification test; GPAQ, global physical activity questionnaire; HEI, healthy eating index; PA, physical activity; PSQI, pittsburgh sleep quality index.

* Differences between baseline and 1-year follow-up were obtained with McNemar test; Bold indicates a *p*-value < 0.05.

## 4 Discussion

This observational study aimed to examine changes in lifestyle behavior in physiotherapy students after one year at the *Universidad de Las Americas* in Quito, Ecuador. The main findings of our study were, at the end of the first year of university, most students transitioned to unhealthy lifestyle behaviors characterized by an increase in weight and BMI, a decrease in the level of physical activity, low adherence to 24-hour movement guidelines, more sleep disturbance, a poor diet quality, and harmful alcohol consumption.

Our findings confirm the observations that the trend of unhealthy lifestyles and poor dietary habits is increasing at the end of the first year of university (5, 7, 9). Although, most of these studies were not conducted in health students. This could be a factor that modifies the results due to these students' having greater knowledge of lifestyle behaviors (34). Despite, this trend can be attributed to numerous factors, including peer pressure, being away from home for a considerable number of hours, the pressure of studying, accessibility of fast foods, and the limited choices of healthy foods on university campuses, in addition to the influence of social media (3, 35). Accordingly, universities should implement prevention and promotion strategies that modify university settings to improve healthy lifestyle behaviors among their students (6).

Regarding weight gain, two meta-analyses reported that university students gain an average of 1.75 kg and 1.55 kg during their first year, respectively (10, 11). Likewise, another meta-analysis reported that first-year university students, on average, gained 1.36 kg over a period of six weeks to eight months. Indeed, 60.9% of students gained weight, and within those, the average weight gained was 3.38 kg (8). It is important to highlight that these meta-analyses do not include studies conducted in Latin America. Our findings agree with a study conducted in Faculty of Health students of one private University of Chile, which reported that students gain an average of 5.9 kg at the end of their first year of university (9).

There is consensus that a critical time for the increase in the prevalence of overweight/obesity appears to be in later adolescence and early adulthood (36). During this period, there is a significant decrease in the level of physical activity (37). According with our findings, another study showed a decrease of 147.9 total METs at one year of follow-up from the upon admission to university (9). Given the low levels of physical activity and unhealthy eating, it is not surprising that the prevalence of overweight/obesity has reached epidemic proportions in young adults in the last decade (38). At the end of the follow-up, the students significantly increased the proportion of overweight/obesity and decreased levels of physical activity. According to our findings, a systematic review reported that insufficient activity is a serious health concern among university students (39). Despite this, there are no studies that have analyzed whether the levels of physical inactivity in students belonging to health careers are similar to those in other careers or faculties. We think that our results could be influenced because our cohort were physiotherapy students, however, similar studies are lacking to compare our findings.

Adequate sleep quality and duration have a critical role in optimal physical health, immune function, mental health, and cognition (40). Unfortunately, attending university is often associated with insufficient sleep (41). After one year of university, 79 students (79%) of our sample had significant sleep disturbance. Usually, factors such as reduced parental support, increased stress from academic loads, and unhealthy lifestyle behaviors result in sleep disorders in this population (42). There are also no studies that have compared sleep quality between students from health faculties and students from other faculties. We could hypothesize that faculties of health students having a better awareness of sleep health and therefore having better sleep habits, which could improve their sleep quality in some degree. However, further research are needed to close this knowledge gap.

Currently, no published studies have evaluated changes in adherence to the overall 24-hour movement guidelines among Latin American university students. Despite this, one study conducted in eight Latin American countries reported that the proportion of adults (aged 18 to 64 years old) who met all three of the 24-hour movement guidelines was low (1.6%) (43). Interestingly, this study showed that sociodemographic factors such as being a woman and having a middle or high education level were associated with a lower probability of meeting all three guidelines (43). Consistent with these findings, our study reports that only six students (6%) met the overall 24-hour movement guidelines. Unfortunately, the lack of similar published studies of Latin American university students prevents us from comparing our findings within this geographical location.

University is often associated with students having more autonomy over their dietary choices. Specifically, factors such as cost, skipping meals, inadequate variety of foods, snacking, and frequent consumption of fast foods increase students' risk of poor health (38). Previous studies have shown that students' diets are characterized by low consumption of fresh fruits and vegetables, monounsaturated fatty acids, polyunsaturated fatty acids, and fish, coupled with increased intake of sugar, alcohol, and fast food (2, 5, 7, 44). Consistent with these findings, our study revealed that 80 students (80%) in the sample exhibited a need for improvements in diet quality and reported engaging in unhealthy eating habits.

In our study, we report a total of 30 students (30%) with harmful alcohol consumption. Consistent with our findings, another study of university students in Ecuador reported that the prevalence of harmful alcohol consumption, above the cutoff score of eight in the AUDIT questionnaire, was high. Almost 50% of male students and 25% of female students reported harmful alcohol consumption (33). The rates of harmful alcohol consumption reported in our study are similar to Europe and South America (where rates range between 23% and 33% for males and 10% and 22% for females) (45).

### 4.1 Limitations and strengths

This study has several limitations. First, as this was an observational study, it did not include a comparison group of young adults who did not attend university but who may also have significant lifestyle changes. Second, these findings should be interpreted with caution, since this research does not allow establishing causality and we also do not have a half-time or medium-term measurement. Third, self-report questionnaire was used for the assessment physical activity level, which can introduce response bias due to over- or underestimation of actual activity levels. Fourth, our study was performed only on students at a private university physiotherapy school. An important factor to consider is that majority of healthcare students have major knowledge about good lifestyles. This may mean that when carrying out the different surveys their perception could be influenced by their acquired knowledge considering cognitive discordance. Therefore, our findings cannot be generalized to all university students. Conversely, this is the first study in Ecuadorian university students with 1-year follow-up to evaluate adherence to 24-hour movement guidelines, diet, and alcohol consumption.

## Conclusion

At the end of the first year of university, most students showed unhealthy lifestyle behaviors characterized by an increase in weight and BMI, a decrease in the level of physical activity, low adherence to 24-hour movement guidelines, more sleep disturbance, poor diet quality, and harmful alcohol consumption. This indicates a need to address this problem during this critical period for developing interventions to improve lifestyle behaviors and prevent the occurrence of non-communicable chronic diseases. In the university, there is significant scope to implement specific strategies to foster healthier lifestyles in their students. However, more research is needed in Latin American universities.

## Data Availability

The raw data supporting the conclusions of this article will be made available by the authors, without undue reservation.
